# Identification of novel potential biomarkers in infantile hemangioma via weighted gene co-expression network analysis

**DOI:** 10.1186/s12887-022-03306-1

**Published:** 2022-05-01

**Authors:** Bin Xie, Xiongming Zhou, Jiaxuan Qiu

**Affiliations:** 1 Department of Vascular Surgery, The First Affiliated Hospital of Nanchang University, No. 17 Yongwai zheng street, 330006 Nanchang, Jiangxi People’s Republic of China; 2 Department of Oral and Maxillofacial Surgery, The First Affiliated Hospital of Nanchang University, No. 17 Yongwai zheng street, 330006 Nanchang, Jiangxi People’s Republic of China

**Keywords:** Infantile hemangioma, WGCNA, Angiogenesis

## Abstract

**Background:**

Infantile hemangioma (IH) is the most common benign tumor in children and is characterized by endothelial cells proliferation and angiogenesis. Some hub genes may play a critical role in angiogenesis. This study aimed to identify the hub genes and analyze their biological functions in IH.

**Methods:**

Differentially expressed genes (DEGs) in hemangioma tissues, regardless of different stages, were identified by microarray analysis. The hub genes were selected through integrated weighted gene co-expression network analysis (WGCNA) and protein–protein interaction (PPI) network. Subsequently, detailed bioinformatics analysis of the hub genes was performed by gene set enrichment analysis (GSEA). Finally, quantitative real-time polymerase chain reaction (qRT-PCR) analysis was conducted to validate the hub genes expression in hemangioma-derived endothelial cells (HemECs) and human umbilical vein endothelial cells (HUVECs).

**Results:**

In total, 1115 DEGs were identified between the hemangiomas and normal samples, including 754 upregulated genes and 361 downregulated genes. Two co-expression modules were identified by WGCNA and green module eigengenes were highly correlated with hemangioma (correlation coefficient = 0.87). Using module membership (MM) > 0.8 and gene significance (GS) > 0.8 as the cut-off criteria, 108 candidate genes were selected and put into the PPI network, and three most correlated genes (APLN, APLNR, TMEM132A) were identified as the hub genes. GSEA predicted that the hub genes would regulate endothelial cell proliferation and angiogenesis. The differential expression of these genes was validated by qRT-PCR.

**Conclusions:**

This research suggested that the identified hub genes may be associated with the angiogenesis of IH. These genes may improve our understanding of the mechanism of IH and represent potential anti-angiogenesis therapeutic targets for IH.

## Introduction

Infantile hemangioma (IH) is the most common benign tumor of the vascular endothelium in children with a prevalence of 4–10% of children younger than 12 months [1–3]. It is most frequently found in the head and neck around 60% of the cases, followed by the trunk and extremities. The main risk factors of IH include premature delivery, low birth weight, female, pre-eclampsia and placental anomalies [4, 5]. It is characterized by an initial rapid proliferation during infancy (proliferation phase), followed by gradual spontaneous regressed over 1–5 years (involution phase), and continual improvement until 6–12 years of age (involuted phase) [6]. The pathology of IH is characterized by the active proliferation of hemangioma-derived endothelial cells (HemECs), which destroys the balance between mitosis and apoptosis and then leads to vasculogenesis and angiogenesis. In clinical, IH is typically a single lesions and can spontaneously regress over time without any complications [7, 8]. However, almost 10% to 15% of IH can cause obstruction, ulcerative, disfiguring and life-threatening complications and sequelae, which need to be treated in time and effectively [8]. Currently, the treatment for IH mainly includes drug therapy (corticosteroids, propranolol, ACEI), surgical operation, injection of local sclerosing agents, laser intervention, etc. [9, 10]. At present, the principle of drug treatment and the pathogenesis of IH are not fully understood, and the clinical treatment probability of IH is reduced due to the emergence of drug resistance, recurrence and rebound cases. Therefore, it is very important to understand the pathogenesis and drug action mechanism of IH in detail and to find the potential target of IH treatment [11, 12].

At present, the detailed etiology and specific mechanisms of the development of IH remain unclear. So we need a better understanding of the pathogenesis of IH that will provide some innovative ideas for exploring more effective treatment strategies. Weighted gene co-expression network analysis (WGCNA) is a widely used method to build co-expression network and identify biomarkers through pairwise correlation matrices [13]. Exclusively based on co-expression analysis will better represent genes with a small effect size acting together [14]. WGCNA provides a systems-level insight into the signaling networks that may be associated with a phenotype of interest [15]. From our study, we identified the hub genes and molecular mechanisms of IH and normal tissue by identifying differences in gene expression, with the hope that those biomarkers will guide medical decisions towards optimal treatments. Our integrative strategy and analysis revealed that several novel differential genes were involved in the occurrence and development of IH.

## Materials and methods

### GEO microarray data

We searched for the microarray data of IH in the Gene Expression Omnibus (GEO) database (https://www.ncbi.nlm.nih.gov/geo/). We got the gene expression profiles of GSE127487 [16], which included 18 samples of hemangiomas without treatment, 5 samples of normal skin tissue without treatment, and 5 samples of hemangiomas with propanolol treatment. In our present study, 5 hemangiomas samples with propanolol treatment were excluded. For the specific data preprocessing of GSE127487, we used the Illumina HumanHT-12 V4.0 platform, retrained the probes with detection *p*-value < 0.05 in more than 23 samples, and normalized by the limma package [17]. After excluding the probes that were unable to be annotated, we combined the probes annotated with the same genes using the median method.

### Identification of DEGs

Differentially expressed genes (DEGs) in the selected modules between hemangiomas samples and normal skin tissue samples were identified using the Limma R package [17]. Subsequently, we convert the gene probe ID into a gene symbol code using R software. Then, the fold change (FC) of gene expression between hemangiomas samples and normal skin tissue samples was obtained, and correction for multiple testing was performed with the Benjamini–Hochberg method. DEGs were identified with a |log FC|> 1 and *p*-value < 0.05. And heatmaps were generated using the pheatmap package.

### Functional annotation and pathway enrichment analysis

Gene Ontology (GO) functional and Kyoto Encyclopedia of Genes and Genomes (KEGG) pathway enrichment analysis were performed to explore the potential biological roles of DGEs [18]. GO analysis included categories of biological processes (BP), cellular component (CC), and molecular function (MF). Pathway enrichment analysis is a functional analysis that maps genes to KEGG pathways. And *p*-value < 0.05 was set as the cut-off point.

### WGCNA construction and module selection process

The WGCNA of DEGs was performed using the WGCNA R software package [19] to construct the co-expression network. Firstly, DEGs were selected from the GSE127487 gene expression datasets. We calculated the soft threshold value based on the “sft” function. Relationships between one gene and all the other genes in the analysis were incorporated, and the adjacency matrix was transformed into the topological matrix (TOM). Subsequently, a hierarchical clustering analysis of genes was performed using 1-TOM as the distance measure [20]. To acquire a small number of large modules, modules were detected using a dynamic tree cut algorithm with a minimum cut height of 0.90 and a minimum module size of 30. Furthermore, module preservation between the two datasets was measured using the specific function of the WGCNA software package [19]. After the clinical information was imported into the co-expression network, the module eigengene (ME) was calculated. ME is representative of the gene expression profiles in a module, illustrating the average expression level of genes in the module. The module could serve as a candidate if it had a high correlation value between the ME valve and the clinical trait. We measured the correlation between the modules and clinical traits of samples by Pearson’s correlation analysis. Only the modules with both high preservation and high correlation were exported for further analysis [20].

### Protein–Protein Interaction (PPI) network construction

We imported the predicted genes into STRING (https://string-db.org/cgi/input.pl) to construct a PPI network. The PPI network was visualized in Cytoscape 3.7.1 software. Functional module analysis was conducted using the “mcode” function to cluster a given network to a densely connected territory based on topology. The selection criteria were established as follows: degree cut-off = 2, node score cut-off = 0.2, k-score = 2 and max depth = 100 [21]. Then the key genes were selected based on the score.

### Gene set enrichment analysis (GSEA)

The GSEA was used to associate the potential gene signature sets by comparing the hemangiomas samples with normal skin tissue samples [22]. The expression profile of DEGs in selected modules of GSE127487 was exported and analyzed with version 4.1.0 of GSEA. The false discovery rate (FDR) is the primary statistic for examining gene set enrichment results, and the nominal *p*-value estimates the statistical significance of the enrichment score. Therefore a gene set with an FDR < 0.25 and a nominal *p*-value < 0.05 was considered to be significantly enriched.

### Quantitative Real-Time PCR (qRT-PCR) Validation in HemECs

Total RNA was extracted from HemECs and HUVECs using RNAiso Plus (TaKaRa, Japan) and the cDNA was reverse-transcribed using SweScript RT I First Strand cDNA Synthesis Kit (With gDNA Remover) (Servicebio, China). qRT-PCR analysis was performed using 2 × SYBR Green qPCR Master Mix (None ROX) (Servicebio, China) according to the manufacturer’s protocol. The relative expression levels of mRNAs of the hub genes were quantified using the 2^−ΔΔCt^ method and normalized to GAPDH expression. All the primers used in this study are listed as follow:APLN-F:5’-TGCTCTGGCTCTCCTTGAC-3’,APLN-R:5’-GCCCATTCCTTGACCCTCT-3’;APLNR-F:5’-TCTGGTGCTCTGGACCGTGT-3’,APLNR-R:5’-TCCCAAAGGGCCAGTCATAGT-3’;TMEM132A-F:5’-GCCTTCTGTGGGAAGGATGTG-3’,TMEM132A-R:5’-TCAGAGAGGAGTTGGCAGGT-3’;GAPDH-F:5’-GGAAGCTTGTCATCAATGGAAATC-3’,GAPDH-R:5’-TGATGACCCTTTTGGCTCCC-3’. The differences between the two groups were compared by the Student’s t-test. *p*-value < 0.05 (two-sided) was considered significant.

## Results

### Identification of DEGs between IH and normal skin tissues

**Fig. 1:**
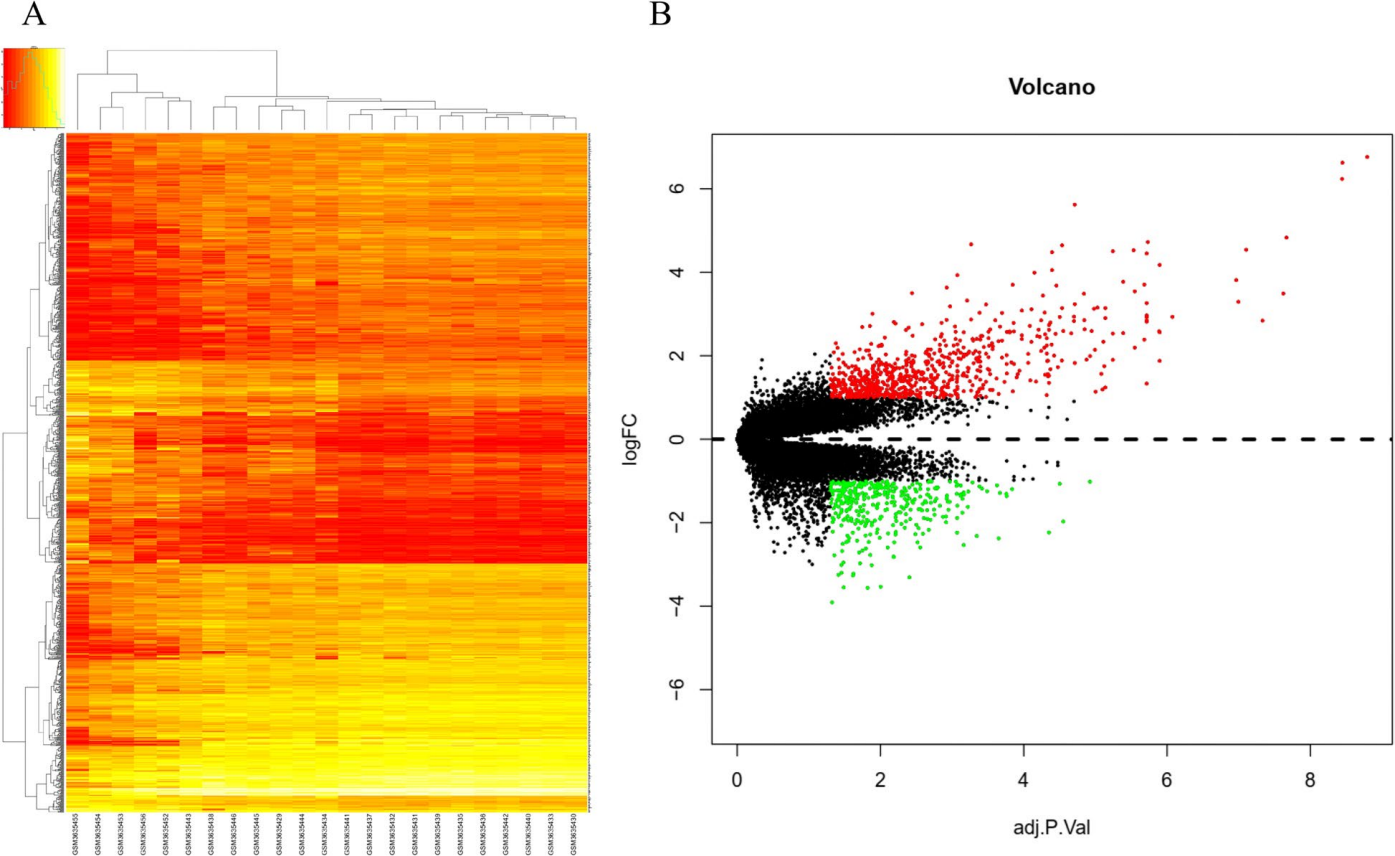
Identification of differentially expressed genes (DEGs) between hemangiomas tissues and normal skin tissues. **A** Heatmap showed that hemangiomas samples were separated from normal samples using hierarchical cluster analysis; (**B**) Volcano plot of the DEGs

Based on the cut-off criteria of |log FC|> 1 and *p*-value < 0.05, 1115 DEGs were identified between 18 samples of hemangiomas without treatment and 5 samples of normal skin tissue without treatment from the GSE127487 using the “limma” package, which includes 754 upregulated genes and 361 downregulated genes. For the difference analysis, hierarchical cluster analysis revealed that the IH samples were obviously separated from normal samples in the heatmaps (Fig. 1A), indicating the reliability of the DEGs. A volcano plot of the 1115 DEGs was shown in Fig. 1B.

### GO and KEGG functional enrichment analysis

**Fig. 2:**
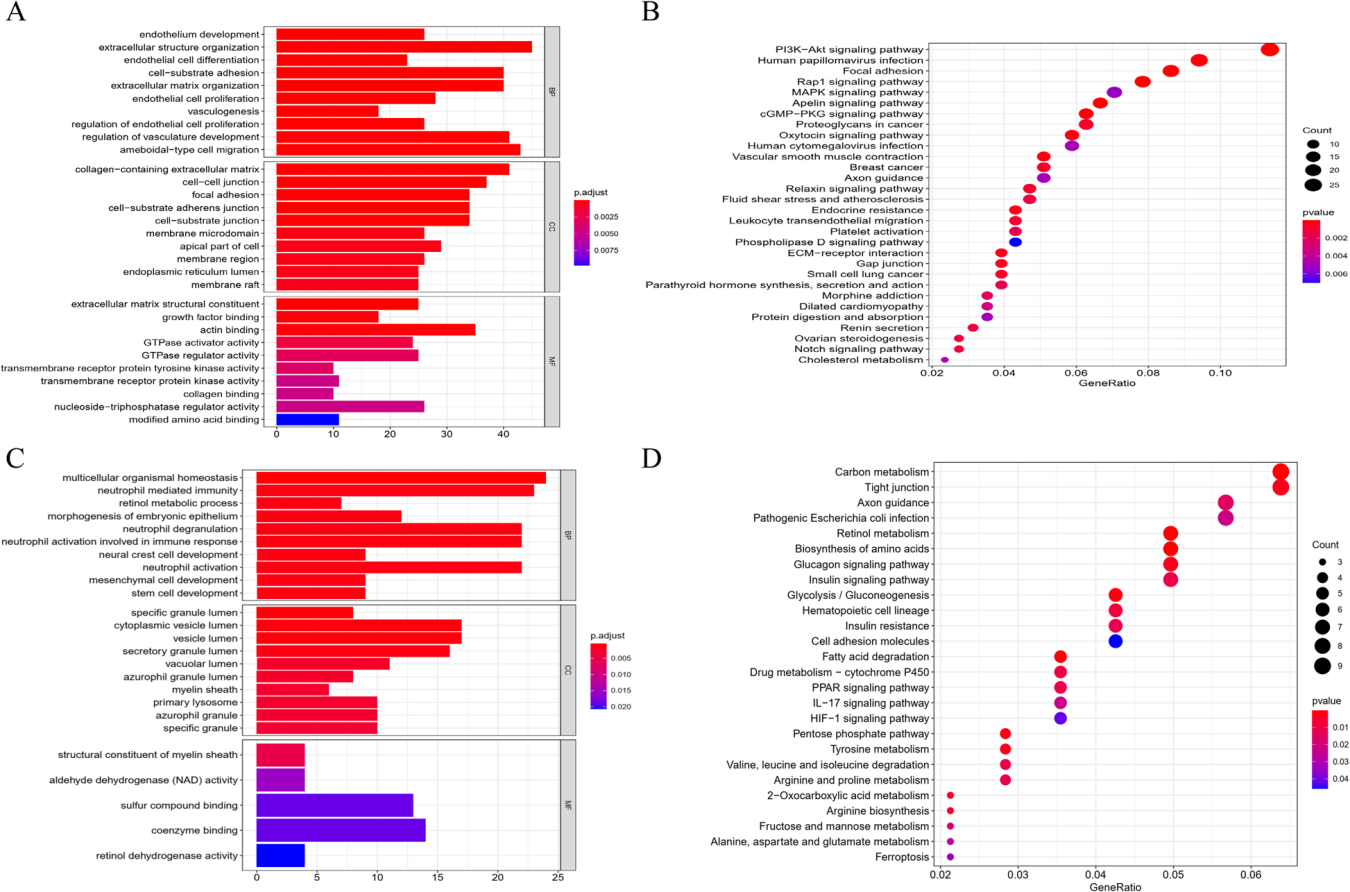
Gene Ontology (GO) functional annotation and pathway enrichment analysis of identified differentially expressed genes (DEGs). GO terms including include biological progress (BP), cellular component (CC), and molecular function (MF). The X-axis represents the GeneRatio and the Y-axis represents the names of Kyoto Encyclopedia of Genes and Genomes (KEGG) terms by KEGG analysis. The red and blue points represent high and low false discovery rate values. **A** Enrichment result of upregulated genes by GO analysis; (**B**) Pathway enrichment analysis result of upregulated genes by KEGG; (**C**) Enrichment result of downregulated genes by GO analysis; (**D**) Pathway enrichment analysis result of downregulated genes by KEGG

The upregulated and downregulated genes were analyzed by the database of Annotation, Visualization and Integration Discovery. The upregulated genes were significantly enriched in several GO terms, including endothelium development, extracellular structure organization, endothelial cell proliferation, endothelial cell differentiation, vasculogenesis, regulation of vasculature development in BP and cell–cell junction in CC, and extracellular matrix structural constituent, growth factor binding in MF (Fig. 2A). The downregulated genes were also enriched in several GO terms, such as multicellular organismal homeostasis, stem cell development, neutrophil degranulation, vesicle lumen, structural constituent of meylin sheath, and coenzyme binding (Fig. 2C). Based on the KEGG pathway analysis, the upregulated genes were enriched in multiple signaling pathways, including PI3K-Akt signaling pathway, Focal adhesion, MAPK signaling pathway, Apelin signaling pathway, Notch signaling pathway, and ECM receptor interaction (Fig. 2B). In contrast, the downregulated genes were enriched in HIF-1 signaling pathway and glycolysis/ gluconeogenesis (Fig. 2D).

### WGCNA analysis

**Fig. 3:**
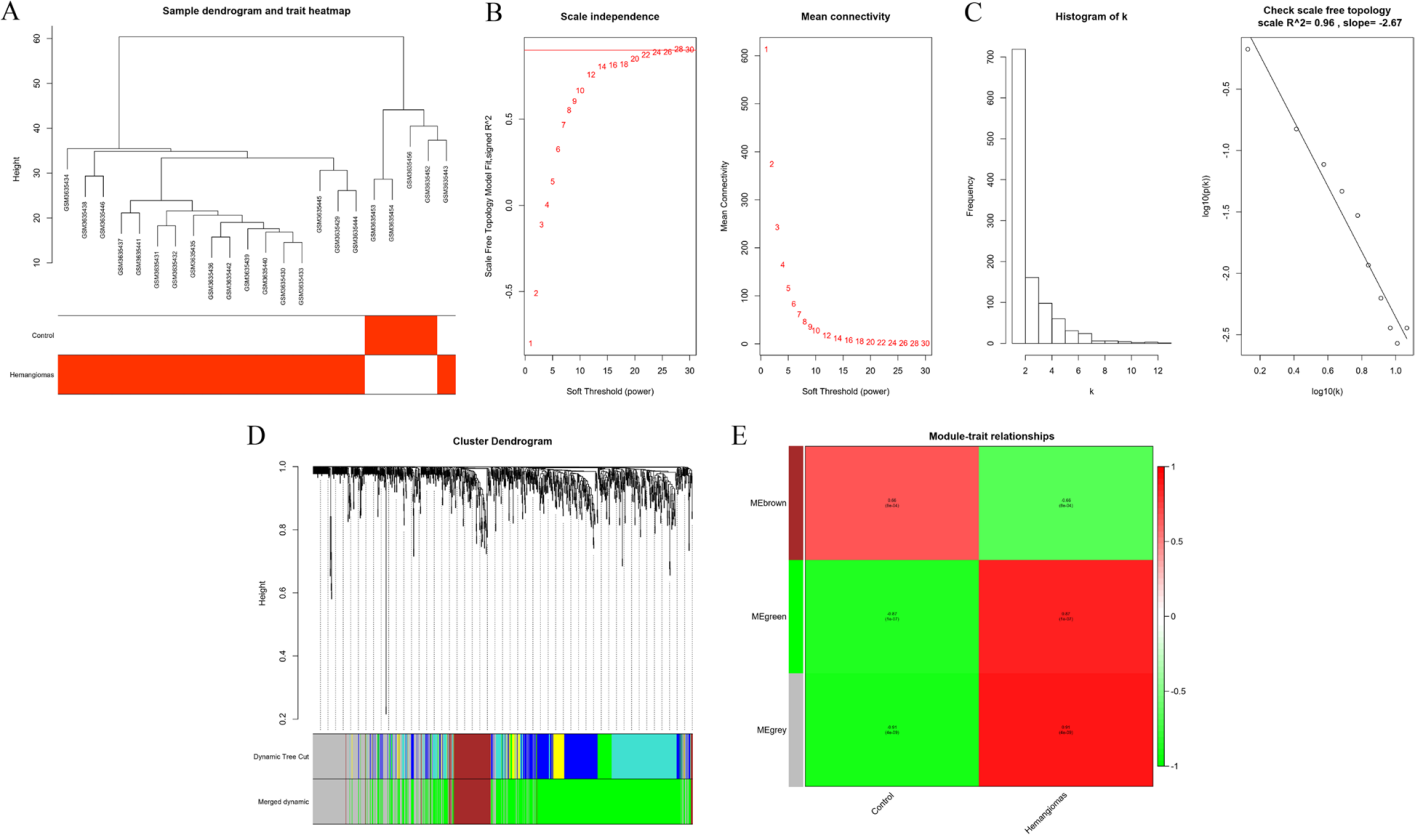
Weighted gene co-expression network analysis (WGCNA) analysis of differentially expressed genes (DEGs). **A** Sample cluster analysis based on RNA data from the GSE127487 database; (**B**) Analysis of network topology for various soft-threshold powers; (**C**) The adjacency matrix was defined using soft-thresholds with β = 28 through check scale-free topology; (**D**) Dendrogram of all DEGs were clustered with dissimilarity according to topological overlap (1-TOM); (**E**) Associated relationships between modules and traits

Our study attempted to identify co-expression modules with high preservation of the DEGs in the GSE127487 and strong IH association using the R package “WGCNA”. Sample cluster analysis based on RNA data from the GSE127487 database showed that there was an outlier sample (GSM3635455), which was eliminated in the next analysis (Fig. 3A). A scale-free network was constructed using soft-thresholding power set to β = 28 (scale free R^2^ = 0.96) (Fig. 3B, C). Two modules were identified based on the mean linkage hierarchical clustering (Fig. 3D) and soft-thresholding power (β = 28). Genes that did not belong to the green or brown modules were placed into the gray module and not used for subsequent analysis. WGCNA analysis indicated that green module eigengenes were highly correlated with hemangioma, and the correlation coefficient was 0.87 (Fig. 3E). And 671 green module eigengenes were identified for subsequent analysis.

### Protein–Protein Interaction (PPI) network construction

**Fig. 4:**
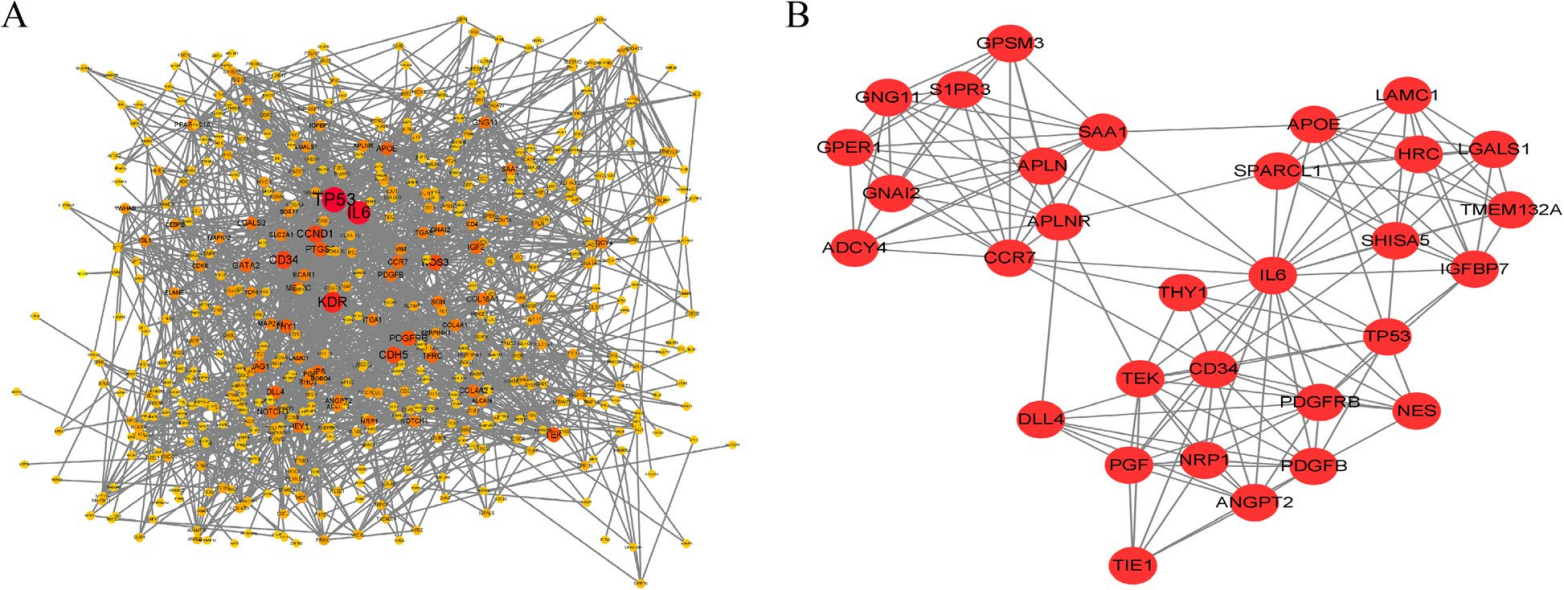
Protein–Protein Interaction (PPI) network construction. **A** PPI network of 671 green module eigengenes. The red the color, the higher the connectivity of genes. **B** PPI network of 31 key genes

In our WGCNA analysis, the green module exhibited the highest correlation with hemangioma (correlation coefficient = 0.87) (Fig. 3E), and so was identified as the hub module eigengenes, which contained 671 genes. The hub module eigengenes was used to construct the Protein–Protein Interaction (PPI) network using the Cytoscape 3.7.1 software (Fig. 4A). And then, we obtained 31 key genes with the highest connectivity for further analysis through the “mcode” function, which we also showed (Fig. 4B).

### Identification of Hub genes by Venn analysis

**Fig. 5:**
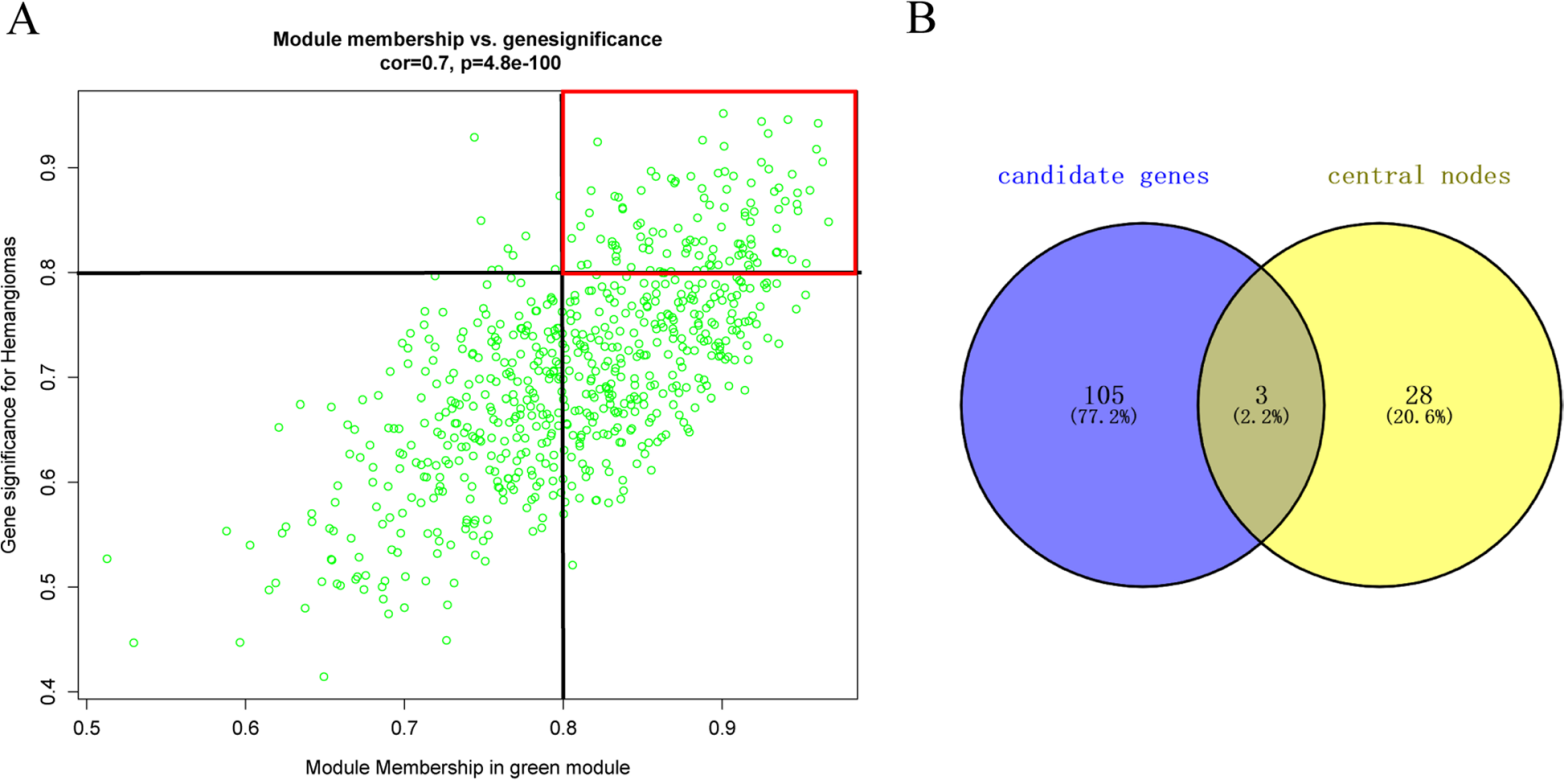
Identification of Hub genes by Venn analysis. **A** A scatter plot of the genes in the green module. Each green dot represents a gene, and dots within the red box indicate genes of module membership > 0.8 and gene significance > 0.8. **B** Hub genes were identified based on overlap within two PPI networks

Genes highly related to the green module were considered potential key genes associated with hemangioma development. Using module membership (MM) > 0.8 and gene significance (GS) > 0.8 as the cut-off criteria, 108 candidate genes were selected (Fig. 5A). 31 key genes were identified by using the “mcode” function from the PPI network (Fig. 4B). Finally, both selected 3 genes were designated as hub genes (APLN, APLNR, TMEM132A) using venn analysis (Fig. 5B).

### Gene Set Enrichment Analysis

**Fig. 6:**
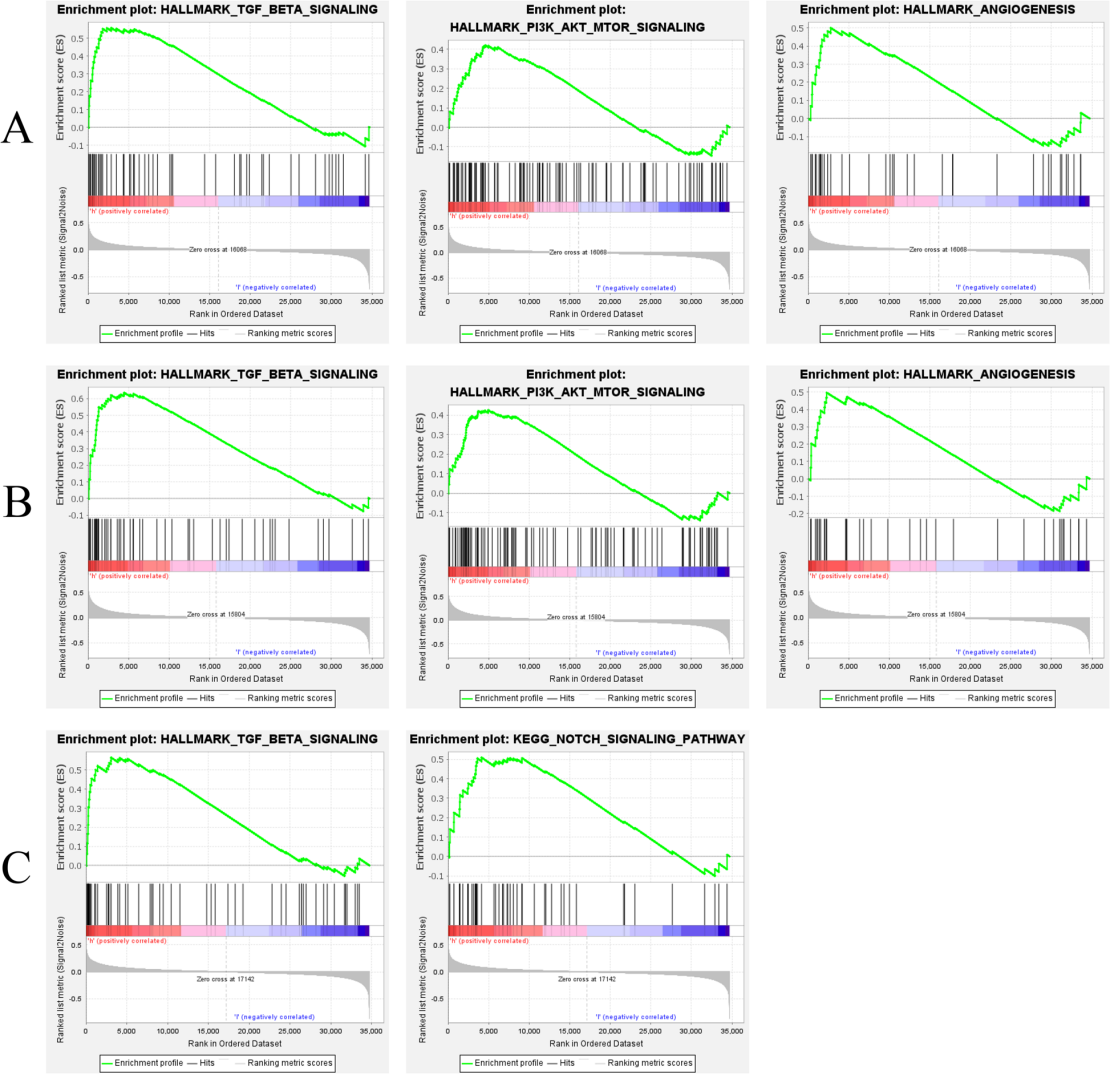
The relationship between three hub genes and hemangiomas. **A** GSEA analysis of APLN shows that APLN is related to the angiogenesis process and signaling pathway in hemangiomas. **B** GSEA analysis of APLNR shows that APLNR is related to the angiogenesis process and signaling pathway in hemangiomas. **C** GSEA analysis of TMEM132A shows that TMEM132A is related to TGF-β and Notch signaling pathway

GSEA analysis was performed to explore the possible mechanisms of three hub genes in hemangioma for further clarification. For APLN phenotype, GSEA analysis suggested that APLN was significantly enriched at nominal *p*-value < 0.05 and FDR < 25% in gene sets such as Hallmark TGF-β signaling, Hallmark PI3K-Akt-mTOR signaling and Hallmark angiogenesis (Fig. 6A). APLNR was also significantly enriched at Hallmark TGF-β signaling, Hallmark PI3K-Akt-mTOR signaling and Hallmark angiogenesis (Fig. 6B). TMEM132A was significantly enriched in Hallmark TGF-β signaling and KEGG Notch signaling pathway (Fig. 6C).

### Validation by qRT-PCR of three hub genes

**Fig. 7:**
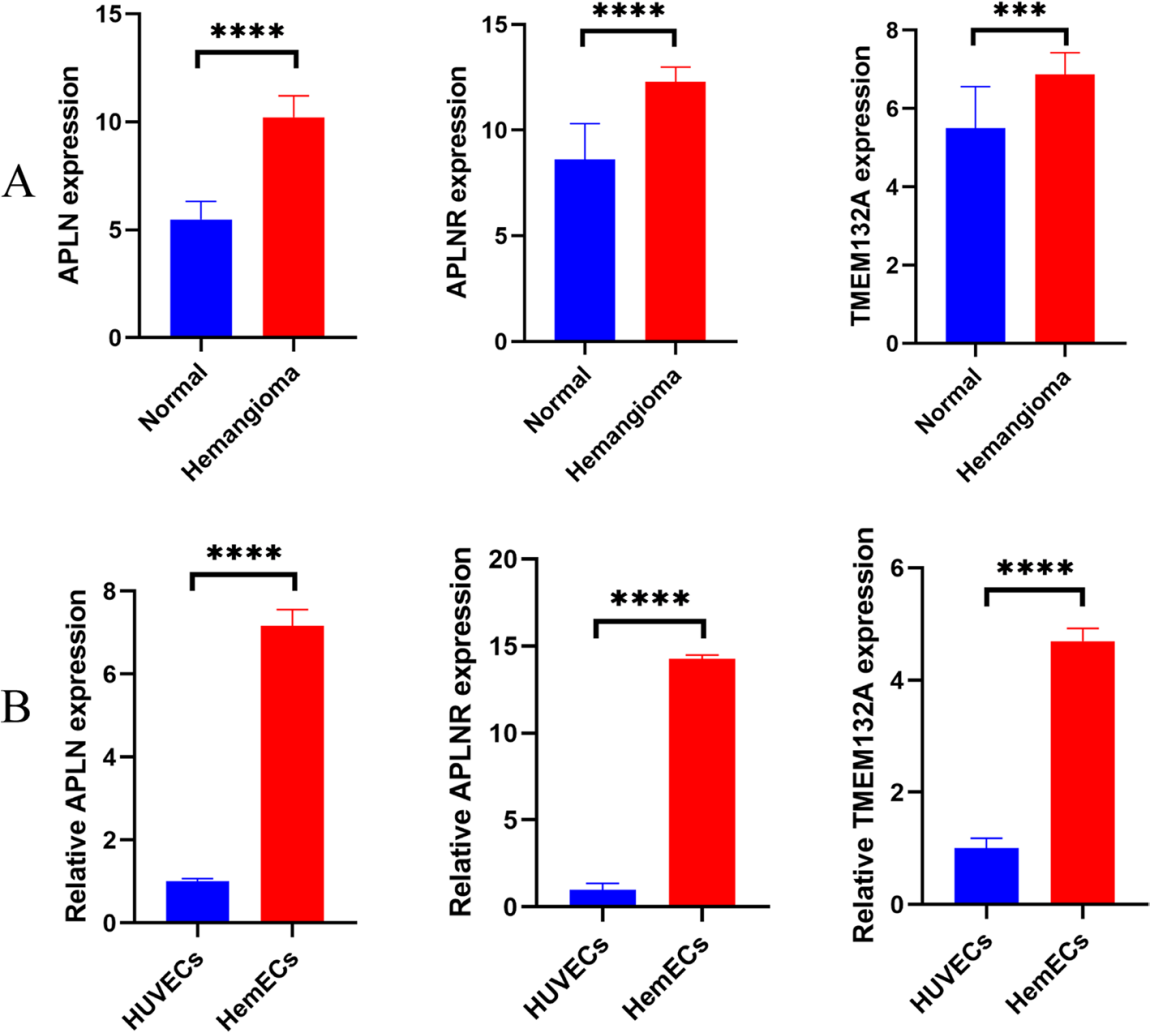
Validation of three hub genes by qRT-PCR between HemECs and HUVECs. **A** The average expression level of three hub genes in the GSE127487 dataset between hemangioma tissues and normal skin tissues. ∗  ∗  ∗  ∗ *P* < 0.0001, ∗  ∗  ∗ *P* < 0.001. **B** All samples were normalized to the relative expression levels of GAPDH, and the relative expression levels of each gene between HemECs and HUVECs were analyzed using the 2^−△△Ct^ method. ∗  ∗  ∗  ∗ *P* < 0.0001

To validate microarray results, the relative expression levels of three hub genes in HemECs and HUVECs were determined using qRT-PCR. The verification result showed that the relative expression levels of three hub genes were significantly increased in HemECs (*p* < 0.05) (Fig. 7B). All validations are consistent with the microarray data and analytical results in this study (Fig. 7A).

## Discussion

IH is one of the most common benign tumors in children. Although most IH can spontaneously regress without any treatment. However, there are still some hemangiomas that will not regress and further aggravate, which makes them difficult to treat in clinical. Therefore, it is essential for finding a new treatment for IH. This study tried to find some potential therapeutic targets for hemangiomas. Firstly, we get the gene expression profiles of GSE127487 and select the DEGs through the limma R package. Then, the DEGs were significantly enriched in several prosess, including endothelium development, endothelial cell proliferation, endothelial cell differentiation, and vasculogenesis in our study. Finally, three genes (APLN, APLNR, TMEM132A) were identified as hub genes, which were consistently highly expressed in hemangioma tissues, regardless of different stages, through integrated WGCNA and PPI network analysis of DEGs.

APLN encoded preproprotein is proteolytically processed into biologically active C-terminal peptide fragments, which activate different tissue specific signaling pathways that regulate diverse biological functions, including fluid homeostasis, insulin secretion, cardiovascular function, endothelium development and tumor angiogenesis. APLN was initially identified as a small secreted peptide due to its inotropic activity [23]. Subsequently, APLN was also described as a tip cell-enriched gene [24]. Preliminary studies demonstrated that APLN signaling is necessary to promote sprouting angiogenesis during embryonic development [25, 26]. Kidoya’s studies showed that APLN knockout (APLN-KO) mice exhibit mild vascular effects such as reduced vessel diameter, compared to wild-type controls, while APLN overexpressed mice developed enlarged but stable vessels with reduced vascular permeability [27, 28]. Recently a study reported that APLN signaling is required to boost endothelial metabolic activity during angiogenic sprouting in zebrafish [29]. Furthermore, this study showed that APLN signaling acts downstream of Notch signaling, where it is required for Notch-controlled angiogenesis [29]. Consequently, APLN-creER reporter mice receiving adequate tamoxifen stimulation allow them to readily differentiate sprouting endothelium from stabilized vasculature during development and in pathology [30]. All these studies showed that APLN was most strongly expressed in endothelial cells and APLN signaling was associated with sprouting angiogenesis. Interestingly, our study found that APLN was highly associated with endothelial cell proliferation, angiogenesis and enriched in PI3K-Akt-mTOR signaling, TGF-β signaling, Notch signaling pathway by GSEA analysis. These biological processes and signaling pathways have been confirmed to play an important role in the occurrence, development and angiogenesis of hemangiomas [31–38]. Meanwhile, our verification result showed that the relative expression level of APLN was significantly increased in HemECs. However, specific biological functions of APLN have not been studied in IH and have potential value for further research as a novel biomarker of hemangioma.

APLNR encodes a G protein-coupled receptor, which is related to the angiotensin receptor but is actually an APLN receptor that inhibits adenylate cyclase activity and plays a counter-regulatory role against the pressure action of angiotensin-II by exerting a hypertensive effect. It functions in the cardiovascular and central nervous systems, glucose metabolism, embryonic and tumor angiogenesis. Devic’s studies showed that APLNR was continuously expressed throughout the developing vasculature while its ligand APLN was localized to the leading edge of APLNR-positive vessels during embryogenesis [39, 40]. The close structural relationship of APLNR to CXC chemokine receptors suggests that APLN acts as a chemokine or chemotactic signal for endothelial cells [41]. Lacking of APLNR in frog embryos was reported to exhibit vascular effects such as decreased endothelial cell proliferation, reduced vascular outgrowth, smaller vessel diameter, and defects in the alignment of arteries and veins [25]. Subsequently, the function of APLN was confirmed for APLNR-expressing endothelial cells in vitro experiments [25, 26, 42, 43]. Therefore, APLN/APLNR pathway plays a key role in several physiological responses, such as endothelium development, metabolic homeostasis, cardiovascular functions, and sprouting angiogenesis. In the present study, APLNR was also highly enriched in TGF-β signaling, angiogenesis, and PI3K-Akt-mTOR signaling. These results suggested that APLNR may be associated with the embryonic and angiogenesis process in IH. Similar to the qRT-PCR result of APLN, the relative expression level of APLNR was also significantly increased in HemECs. It is worth noting that the study of APLNR in IH has also not been reported and has potential research value.

Transmembrane protein 132A (TMEM132A) belongs to the TMEM132 family, composed of five members (TMEM132A-E). TMEM132A was firstly identified as a novel gene expressed in the rat brain during the embryonic and postnatal stages and as a binding protein for the endoplasmic reticulum resident chaperone 78-kDa glucose-regulated protein [44]. Stably TMEM132A expression was found in the rat embryonic brain and several other tissues, suggesting a functional role in the developing brain and perhaps a crucial role in preventing neurodegeneration [44]. In addition, TMEM132A is also reported to regulate the expression of cAMP-induced glial fibrillary acidic protein in rat C6 glioblastoma cells [45]. Subsequently, TMEM132A-knockdown in Neuro2a cells increases serum starvation-induced endoplasmic reticulum stress related apoptosis [46]. Recently, TMEM132A was identified as a novel regulator in regulating the Wnt signaling pathway through interaction with the Wnt ligand transporting protein Wntless [47]. In addition, analysis of the subcellular localization of TMEM132A revealed that TMEM132A is not only localized in the endoplasmic reticulum-Golgi apparatus, but it is also detected at the cell surface, and its N-terminus is exposed to the extracellular space [48]. These results suggest that TMEM132A might play a role in the perception of extracellular signals and cell–cell communication. However, there is no related research report of TMEM132A in hemangioma. Of note, we found that TMEM132A is related to TGF-β signaling and Notch signaling pathway in hemangioma by GSEA analysis, which is conducive to further research.

## Conclusion

In this study, we identified three hub genes (APLN, APLNR, TMEM132A) highly associated with IH based on the integrated WGCNA and PPI network. These hub genes may regulate the development of IH through endothelial cell proliferation, sprouting angiogenesis, and apelin signaling pathway. These genes may improve our understanding of the mechanism of IH and represent potential anti-angiogenesis therapeutic targets for IH.

## Data Availability

The GSE127487 dataset [16] used and analyzed during the current study are available from the GEO datasets was downloaded from https://www.ncbi.nlm.nih.gov/geo/.

## References

[1] Dickison P, Christou E, Wargon O (2011). A prospective study of infantile hemangiomas with a focus on incidence and risk factors. Pediatr Dermatol.

[2] Smolinski KN, Yan AC (2005). Hemangiomas of infancy: clinical and biological characteristics. Clin Pediatr (Phila).

[3] Rotter A, de Oliveira ZNP (2017). Infantile hemangioma: pathogenesis and mechanisms of action of propranolol. J Dtsch Dermatol Ges.

[4] Munden A, Butschek R, Tom WL (2014). Prospective study of infantile haemangiomas: incidence, clinical characteristics and association with placental anomalies. Br J Dermatol.

[5] Auger N, Fraser WD, Arbour L (2017). Pre-eclampsia and risk of infantile haemangioma. Br J Dermatol.

[6] Takahashi K, Mulliken JB, Kozakewich HP (1994). Cellular markers that distinguish the phases of hemangioma during infancy and childhood. J Clin Invest.

[7] Cheng CE, Friedlander SF (2016). Infantile hemangiomas, complications and treatments. Semin Cutan Med Surg.

[8] Csoma ZR, Dalmády S, Ábrahám R (2017). Infantile haemangioma: clinical and demographic characteristics, experiences in the treatment. Orv Hetil.

[9] Grzesik P, Wu JK (2017). Current perspectives on the optimal management of infantile hemangioma. Pediatric Health Med Ther.

[10] Smith CJF, Friedlander SF, Guma M (2017). Infantile hemangiomas: an updated review on risk factors, pathogenesis, and treatment. Birth Defects Res.

[11] Mabeta P (2018). Oncosuppressors and oncogenes: role in haemangioma genesis and potential for therapeutic targeting. Int J Mol Sci.

[12] Qiu M, Qi X, Dai Y (2015). Infantile haemangioma: a complicated disease. Front Biosci (Landmark Ed).

[13] Feltrin AS, Tahira AC, Simões SN (2019). Assessment of complementarity of WGCNA and NERI results for identification of modules associated to schizophrenia spectrum disorders. PLoS ONE.

[14] Chaste P, Klei L, Sanders SJ (2015). A genome-wide association study of autism using the Simons Simplex Collection: Does reducing phenotypic heterogeneity in autism increase genetic homogeneity?. Biol Psychiatry.

[15] Liang W, Sun F, Zhao Y (2020). Identification of Susceptibility Modules and Genes for Cardiovascular Disease in Diabetic Patients Using WGCNA Analysis. J Diabetes Res.

[16] Gomez-Acevedo H, Dai Y, Strub G (2020). Identification of putative biomarkers for Infantile Hemangiomas and Propranolol treatment via data integration. Sci Rep.

[17] Ritchie ME, Phipson B, Wu D (2015). Limma powers differential expression analyses for RNA-sequencing and microarray studies. Nucleic Acids Res.

[18] Shi X, Huang T, Wang J (2018). Next-generation sequencing identifies novel genes with rare variants in total anomalous pulmonary venous connection. EBioMedicine.

[19] Botía JA, Vandrovcova J, Forabosco P (2017). An additional k-means clustering step improves the biological features of WGCNA gene co-expression networks. BMC Syst Biol.

[20] Bi S, Liu R, Shen Y (2020). Bioinformatics analysis of key genes and miRNAs associated with Stanford type A aortic dissection. J Thorac Dis.

[21] Li L, Lei Q, Zhang S (2017). Screening and identification of key biomarkers in hepatocellular carcinoma: evidence from bioinformatic analysis. Oncol Rep.

[22] Subramanian A, Tamayo P, Mootha VK (2005). Gene set enrichment analysis: a knowledge-based approach for interpreting genome-wide expression profiles. Proc Natl Acad Sci U S A.

[23] Szokodi I, Tavi P, Földes G (2002). Apelin, the novel endogenous ligand of the orphan receptor APJ, regulates cardiac contractility. Circ Res.

[24] del Toro R, Prahst C, Mathivet T (2010). Identification and functional analysis of endothelial tip cell-enriched genes. Blood.

[25] Cox CM, D'Agostino SL, Miller MK (2006). Apelin, the ligand for the endothelial G-protein-coupled receptor, APJ, is a potent angiogenic factor required for normal vascular development of the frog embryo. Dev Biol.

[26] Kälin RE, Kretz MP, Meyer AM (2007). Paracrine and autocrine mechanisms of apelin signaling govern embryonic and tumor angiogenesis. Dev Biol.

[27] Kidoya H, Ueno M, Yamada Y (2008). Spatial and temporal role of the apelin/APJ system in the caliber size regulation of blood vessels during angiogenesis. EMBO J.

[28] Kidoya H, Naito H, Takakura N (2010). Apelin induces enlarged and nonleaky blood vessels for functional recovery from ischemia. Blood.

[29] Helker CS, Eberlein J, Wilhelm K (2020). Apelin signaling drives vascular endothelial cells toward a pro-angiogenic state. Elife.

[30] Liu Q, Hu T, He L (2015). Genetic targeting of sprouting angiogenesis using Apln-CreER. Nat Commun.

[31] Wu JK, Adepoju O, De Silva D (2010). A switch in Notch gene expression parallels stem cell to endothelial transition in infantile hemangioma. Angiogenesis.

[32] Adepoju O, Wong A, Kitajewski A (2011). Expression of HES and HEY genes in infantile hemangiomas. Vasc Cell.

[33] Ji Y, Chen S, Xiang B (2016). Jagged1/Notch3 signaling modulates Hemangioma-Derived Pericyte *proliferation and m*aturation. Cell Physiol Biochem.

[34] Boscolo E, Stewart CL, Greenberger S (2011). JAGGED1 signaling regulates hemangioma stem cell-to-pericyte/vascular smooth muscle cell differentiation. Arterioscler Thromb Vasc Biol.

[35] Medici D, Olsen BR (2012). Rapamycin inhibits proliferation of hemangioma endothelial cells by reducing HIF-1-dependent expression of VEGF. PLoS ONE.

[36] Du W, Gerald D, Perruzzi CA (2013). Vascular tumors have increased p70 S6-kinase activation and are inhibited by topical rapamycin. Lab Invest.

[37] Li D, Li P, Guo Z (2017). Downregulation of miR-382 by propranolol inhibits the progression of infantile hemangioma via the PTEN-mediated AKT/mTOR pathway. Int J Mol Med.

[38] Yamashita T, Jinnin M, Makino K (2018). Serum cytokine profiles are altered in patients with progressive infantile hemangioma. Biosci Trends.

[39] Devic E, Paquereau L, Vernier P (1996). Expression of a new G protein-coupled receptor X-msr is associated with an endothelial lineage in Xenopus laevis. Mech Dev.

[40] Devic E, Rizzoti K, Bodin S (1999). Amino acid sequence and embryonic expression of msr/apj, the mouse homolog of Xenopus X-msr and human APJ. Mech Dev.

[41] Saint-Geniez M, Masri B, Malecaze F (2002). Expression of the murine msr/apj receptor and its ligand apelin is upregulated during formation of the retinal vessels. Mech Dev.

[42] Kasai A, Shintani N, Oda M (2004). Apelin is a novel angiogenic factor in retinal endothelial cells. Biochem Biophys Res Commun.

[43] Papangeli I, Kim J, Maier I (2016). MicroRNA 139–5p coordinates APLNR-CXCR4 crosstalk during vascular maturation. Nat Commun.

[44] Oh-hashi K, Naruse Y, Amaya F (2003). Cloning and characterization of a novel GRP78-binding protein in the rat brain. J Biol Chem.

[45] Oh-hashi K, Hirata Y, Koga H (2006). GRP78-binding protein regulates cAMP-induced glial fibrillary acidic protein expression in rat C6 glioblastoma cells. FEBS Lett.

[46] Oh-hashi K, Imai K, Koga H (2010). Knockdown of transmembrane protein 132A by RNA interference facilitates serum starvation-induced cell death in Neuro2a cells. Mol Cell Biochem.

[47] Li B, Niswander LA (2020). TMEM132A, a Novel Wnt Signaling Pathway Regulator Through Wntless (WLS) Interaction. Front Cell Dev Biol.

[48] Oh-hashi K, Koga H, Nagase T (2012). Characterization of the expression and cell-surface localization of transmembrane protein 132A. Mol Cell Biochem.

